# Diagnosis of non-consensus transient ischaemic attacks with focal, negative, and non-progressive symptoms: population-based validation by investigation and prognosis

**DOI:** 10.1016/S0140-6736(20)31961-9

**Published:** 2021-03-06

**Authors:** Maria A Tuna, Peter M Rothwell, Oxford Vascular Study

**Affiliations:** a Wolfson Centre for the Prevention of Stroke and Dementia, Nuffield Department of Clinical Neuroscience, John Radcliffe Hospital, University of Oxford, Oxford, UK

## Abstract

**Background:**

Diagnosis of transient ischaemic attacks (TIAs) can be difficult. There is consensus on classic symptoms (eg, motor weakness, dysphasia, hemianopia, monocular visual loss) but no consensus on several monosymptomatic events with sudden-onset, non-progressive, focal negative symptoms (eg, isolated diplopia, dysarthria, vertigo, ataxia, sensory loss, and bilateral visual disturbance), with much variation in investigation and treatment.

**Methods:**

We prospectively ascertained and investigated all strokes and sudden onset transient neurological symptoms in a population of 92 728 people (no age restrictions) from Oxfordshire, UK, who sought medical attention at nine primary care practices or at the John Radcliffe Hospital, Oxford, UK (Oxford Vascular Study). Patients classified at baseline with minor ischaemic stroke (National Institutes of Health Stroke Score <5), classic TIA, or non-consensus TIA were treated according to secondary prevention guidelines. Risks of stroke (7-day, 90-day, and 10-year risks) and risks of all major vascular events (from the time of first event, and from the time of seeking medical attention) were established by face-to-face follow-up visits and were compared with the risk expected from age and sex-specific stroke incidence in the underlying study population.

**Findings:**

Between April 1, 2002, and March 31, 2018, 2878 patients were identified with minor ischaemic stroke (n=1287), classic TIA (n=1021), or non-consensus TIA (n=570). Follow-up was to Oct 1, 2018 (median 5·2 [IQR 2·6–9·2] years). 577 first recurrent strokes after the index event occurred during 17 009 person-years of follow-up. 90-day stroke risk from time of the index event after a non-consensus TIA was similar to that after classic TIA (10·6% [95% CI 7·8–12·9] *vs* 11·6% [95% CI 9·6–13·6]; hazard ratio 0·87, 95% CI 0·64–1·19; p=0·43), and higher than after amaurosis fugax (4·3% [95% CI 0·6–8·0]; p=0·042). However, patients with non-consensus TIA were less likely to seek medical attention on the day of the event than were those with classic TIA (336 of 570 [59%] *vs* 768 of 1021 [75%]; odds ratio [OR] 0·47, 95% CI 0·38–0·59; p<0·0001) and were more likely to have recurrent strokes before seeking attention (45 of 570 [8%] *vs* 47 of 1021 [5%]; OR 1·77, 95% CI 1·16–2·71; p=0·007). After excluding such recurrent strokes, 7-day stroke risk after seeking attention for non-consensus TIA (2·9% [95% CI 1·5–4·3]) was still considerably higher than the expected background risk (relative risk [RR] 203, 95% CI 113–334), particularly if the patient sought attention on the day of the index event (5·0% [2·1–7·9]; RR 300, 137–569). 10-year risk of all major vascular events was similar for non-consensus and classic TIAs (27·1% [95% CI 22·8–31·4] *vs* 30·9% [27·2–33·7]; p=0·12). Baseline prevalence of atrial fibrillation, patent foramen ovale, and arterial stenoses were also similar for non-consensus TIA and classic TIA, although stenoses in the posterior circulation were more frequent with non-consensus TIA (OR 2·21, 95% CI 1·59–3·08; p<0·0001).

**Interpretation:**

Patients with non-consensus TIA are at high early and long-term risk of stroke and have cardiovascular pathological findings on investigation similar to those of classic TIA. Designation of non-consensus TIAs as definite cerebrovascular events will increase overall TIA diagnoses by about 50%.

**Funding:**

Wellcome Trust, National Institute for Health Research Oxford Biomedical Research Centre, Wolfson Foundation, Masonic Charitable Foundation, and British Heart Foundation.

## Introduction

Up to 25% of strokes are preceded by a transient ischaemic attack (TIA).^1^ The early risk of stroke after a TIA is high, and urgent investigation and treatment are required.^2^, ^3^, ^4^ In particular, treatment with antiplatelets is needed because these drugs substantially reduce the risk of early recurrent stroke.^5^ Diagnosis of TIA usually relies on the patient's description of symptoms and on the ability of clinicians to interpret them correctly.^6^, ^7^, ^8^ However, the high-level definition of TIA as a sudden, focal neurological deficit of presumed vascular origin lasting less than 24 h^8^, ^9^ provides no guidance on which symptoms are likely to be vascular in origin.

Agreement between clinicians regarding diagnosis of TIA is only moderate.^10^, ^11^, ^12^, ^13^, ^14^ Diagnostic criteria of the National Institute of Neurological Disorders and Stroke (NINDS) were developed by expert consensus to aid clinical practice and recruitment into research.^9^ Specifically, these criteria disqualify several sudden onset and focal monosymptomatic events deemed not to be TIAs, including isolated diplopia, dysarthria, vertigo, ataxia, sensory loss, and bilateral visual impairment.^9^ The argument is that these isolated symptoms often have a non-vascular cause,^8^, ^9^ but interobserver agreement in diagnosis between clinicians is poor.^11^, ^15^

**Research in context**

**Evidence before this study**

We did a systematic review of studies of the prognosis of atypical transient ischaemic attacks (TIAs). We searched Embase and MEDLINE databases for articles published in English up to Dec 1, 2019, using the terms “transient ischaemic attack”, “TIA”, “atypical TIA”, “non-focal TIA”, “transient neurological attack”, “non-specific transient neurological symptoms”, and “TIA mimics”. Studies were selected if they included patients with TIA or other transient neurological symptoms without a non-vascular diagnosis. Our search retrieved no previous study of the prognosis of transient, sudden onset monosymptomatic events as a whole. We identified three studies (Dutch trial, Rotterdam Study, and SOS-TIA) in which the risk of stroke and other vascular events was determined after an atypical TIA or transient neurological attack (appendix p 2). In the Dutch trial, 572 patients had atypical symptoms (a mix of positive, negative, sudden, or progressive non-focal symptoms in isolation or combination) and 2555 patients had symptoms of classic TIA. The 5-year risk of stroke after atypical TIA was lower than after classic TIA (hazard ratio 0·6, 95% CI 0·4–0·9). The Rotterdam Study identified 548 patients with transient neurological attacks, with diagnosis based on patients' recall months to years later and on retrospective review of medical records, but this study excluded patients with monosymptomatic events. In the SOS-TIA clinical study, patients with atypical transient isolated symptoms (four of 172) had a similar 1-year risk of major vascular events to patients with isolated typical TIA symptoms (11 of 607).

**Added value of this study**

We did a large, prospective, population-based, longitudinal cohort study of all suspected TIAs and minor strokes, irrespective of age, with near-complete ascertainment of patients seeking medical attention. Events were prospectively classified at baseline as definite classic TIA or non-consensus TIA (sudden onset of isolated non-progressive negative monosymptomatic events) and were similarly investigated and treated according to secondary prevention guidelines.

**Implications of all the available evidence**

We found that non-consensus TIAs had high early and long-term risks of stroke and had cardiovascular pathological findings on investigation similar to those of classic TIA. Our findings indicate that monosymptomatic events should be managed as TIAs and should not be excluded from treatment trials.

Current guidelines on management of TIAs simply refer back to the NINDS consensus for diagnosis,^9^, ^16^ or ignore interobserver reproducibility of clinical diagnosis, and they focus on imaging criteria for minor stroke.^17^, ^18^ However, since most patients with TIA have no acute ischaemia on imaging,^19^, ^20^, ^21^ symptom-based diagnosis remains the mainstay of clinical practice. Most textbooks still reiterate the NINDS criteria for diagnosis of TIA,^22^, ^23^ and patients with non-consensus symptoms of TIA remain ineligible for treatment trials and other research studies (appendix p 3).^3^, ^4^, ^24^, ^25^

Frequently, patients with non-consensus events are not investigated or treated as TIAs in practice. However, in view of the benefits of urgent treatment after TIA,^5^ if stroke risk is increased early after non-consensus TIA, it is vital that patients are not left untreated. Equally, if long-term stroke risk is low, life-long medication should not be prescribed unnecessarily.

Although the long-term risk of stroke after classic symptoms of TIA (including motor weakness, dysphasia, hemianopia, and monocular visual loss) is known to be high,^26^, ^27^ few data have been published on the prognosis of non-consensus events. Long-term risk of stroke and other vascular events is increased after atypical transient neurological events,^28^, ^29^, ^30^ but previous studies have grouped together many different syndromes, typically including events with positive or progressive symptoms (eg, suggestive of transient neurological attacks) as well as non-consensus TIAs.^28^, ^29^ In a previous study, we reported high rates of transient, focal, negative monosymptomatic events in the days preceding posterior circulation strokes on retrospective questioning.^31^ However, since prospective data for prognosis have previously been vital in differentiating other conditions with transient symptoms from TIAs,^32^, ^33^ we set up a prospective study of all acute suspected cerebrovascular events. Here, we report the incidence, clinical characteristics, investigation, and prognosis of non-consensus TIAs (ie, certain monosymptomatic events with sudden onset, non-progressive, focal deficits) compared with classic TIAs (panel), with short-term stroke risk stratified by two potentially confounding factors—delay to presentation^34^ and early antiplatelet treatment.^5^

Panel **Prospective classification of the most common symptoms of classic TIA versus non-consensus TIA used in the Oxford Vascular Study**

**Classic TIA**

*Motor weakness*

Sudden onset of transient motor weakness in one or more body segment (face, arm, hand, leg)

*Dysphasia*

Sudden onset of transient expressive or receptive dysphasia, or both

*Sensory loss*

Sudden onset of transient sensory loss in two or more body segments (face, arm, hand, or leg)

*Hemianopia or quadrantanopia*

Sudden onset of transient visual loss in part of the visual field (homonymous hemianopia or quadrantanopia)

*Monocular visual loss*

Sudden onset of transient monocular visual loss

*Vertigo plus*

Sudden onset of transient vertigo plus other TIA symptoms

*Diplopia plus*

Sudden onset of transient diplopia plus other TIA symptoms

*Dysarthria plus*

Sudden onset of transient dysarthria plus other TIA symptoms

*Ataxia plus*

Sudden onset of transient ataxia plus other TIA symptoms

**Non-consensus TIA**

*Vertigo only*

Sudden onset of new non-recurrent isolated vertigo (with or without nausea or vomiting) not precipitated by head movement or trauma, and without associated ear pain, tinnitus, or hearing loss; cases with non-specific dizziness or light headedness are excluded

*Ataxia only*

Sudden onset of transient unsteadiness of gait without any other cause

*Diplopia only*

Sudden onset of transient isolated binocular double vision without an obvious ocular (eg, retinal detachment) or neuromuscular cause

*Dysarthia only*

Sudden onset of transient isolated slurred speech

*Bilateral decreased vision only*

Sudden onset of transient isolated bilateral visual impairment (excluding hemianopia or quadrantanopia) without associated positive symptoms

*Single segment sensory loss only*

Sudden onset of transient isolated unilateral numbness in only one body segment (face, arm or hand, or leg) without march

TIA=transient ischaemic attack.

## Methods

### Study design and participants

The Oxford Vascular Study (OXVASC) is a prospective population-based study of the incidence and outcome of all cerebrovascular (stroke and TIA), cardiovascular, and peripheral vascular events in a population of 92 728 individuals, irrespective of age, registered with nine primary care practices (roughly 100 primary care doctors) in Oxfordshire, UK (appendix pp 5–6).^35^, ^36^ In the UK, about 99% of residents register with primary care, which holds a life-long record of all consultations and investigations with primary and secondary care providers. The OXVASC population is 94% white European, 3·1% Asian, 1·5% Chinese, and 1·4% Afro-Caribbean and has a broad range of social deprivation.^35^, ^36^

As part of OXVASC, we prospectively ascertained all individuals with stroke and all people who sought medical attention in primary or secondary care with sudden onset transient neurological symptoms. Acute secondary care services to the OXVASC population are provided by one hospital (John Radcliffe Hospital, Oxford, UK). All participating primary care doctors were requested to refer patients who presented with new sudden onset transient neurological symptoms to a daily TIA and stroke clinic, which provided urgent clinical investigation and treatment as part of OXVASC. Annual reminders were sent to primary care staff throughout the study.

Multiple other sources were used to achieve near-complete identification of patients presenting to medical attention with possible TIA or stroke.^35^, ^36^ First, we made daily visits to the emergency department of the John Radcliffe Hospital to identify patients with a diagnosis of TIA or stroke, or with symptoms of a non-consensus TIA. Second, we made daily visits to the hospital's acute stroke unit, neurology wards, and coronary care unit, and via bereavement officers we identified patients brought into the hospital dead or who died soon after arrival. Third, by cross-referral to the study TIA and stroke clinic, we identified patients with possible TIA or minor stroke who were seen at the John Radcliffe Hospital, the eye-hospital emergency department at the John Radcliffe Hospital, or in other clinics at the hospital. Fourth, we regularly searched the John Radcliffe Hospital's computerised diagnostic records for patients with symptoms of TIA or stroke. Fifth, we made regular searches of records of requests for brain or neurovascular imaging at the John Radcliffe Hospital. Finally, we did monthly searches of primary care computer records for vascular diagnoses.

Patients were seen by study doctors as soon as possible after their initial presentation. Written informed consent was obtained from all patients, or assent was obtained from relatives of patients with dementia or receptive dysphasia. Patients who had an event while temporarily away from Oxfordshire were included on their return, but visitors to Oxfordshire not normally registered with a study general practice were excluded.

### Procedures

Using a standard form, we recorded baseline demographic data, risk factors, clinical history, and symptoms, including duration of attack and number of attacks. Severity of stroke was assessed using the National Institutes of Health Stroke Scale (NIHSS).^37^

All cases were reviewed by the study's senior neurologist (PMR) and classified prospectively, usually on the day of ascertainment, as either non-vascular conditions (eg, migraine aura or seizure), stroke, classic TIA, or non-consensus TIA. Patients with transient positive visual or sensory symptoms or progressive symptoms suggestive of transient neurological attacks were excluded from this analysis and will be reported separately. Non-consensus TIA was prospectively subclassified using predefined definitions (panel), as isolated vertigo, isolated ataxia, isolated diplopia, non-dysphasic isolated speech disturbance (slurred speech), isolated bilateral decreased vision, or isolated unilateral sensory symptoms involving only one body part (eg, face, arm, hand, or leg). In view of known differences in prognosis,^23^, ^24^ classic TIAs were subclassified as amaurosis fugax, cerebral TIA involving motor weakness, or cerebral TIA without motor weakness. Stroke was defined according to WHO criteria;^38^ classic TIA and non-consensus TIA were also classified according to the likely vascular territory (anterior, posterior, or uncertain).

Since not all patients with TIA-like symptoms seek medical attention, with some people delaying for several days or weeks,^34^, ^39^ and with the possibility that delays might differ depending on the nature of symptoms, we did not want to bias our analysis by excluding early recurrent strokes that occurred before seeking medical attention. All patients were also, therefore, asked about any sudden onset transient neurological symptoms or stroke-like events that they had experienced over the 90 days before the event for which they first sought medical attention. Any such events were also classified by PMR as either non-vascular conditions, stroke, classic TIA, or non-consensus TIA.

Patients were investigated with blood tests, brain imaging, electrocardiography, and vascular imaging. From 2002 to 2009, CT brain imaging and extracranial carotid doppler ultrasound imaging were first-line investigations, but from 2009 onwards, patients underwent brain MRI with diffusion-weighted imaging or magnetic resonance angiography of the cerebral and extracranial vessels (or CT angiography if contraindicated). From 2011, patients also had transthoracic echocardiography, 5-day ambulatory cardiac rhythm monitoring to detect paroxysmal atrial fibrillation, and transcranial bubble doppler to detect patent foramen ovale. Details of these investigations have been published previously.^40^, ^41^

Patients whose symptoms were found on initial brain imaging to be due to a brain tumour, subdural haematoma, or vascular malformation were classified as TIA mimics and were excluded. However, for this analysis, the initial symptomatic diagnosis of classic TIA, non-consensus TIA, or minor stroke was not altered in light of findings of acute or previous ischaemia on brain imaging or of arterial stenosis on vascular imaging.

Patients with non-consensus TIA, classic TIA, and minor stroke who were referred to the study TIA or stroke clinic were given secondary prevention, including acute antiplatelet treatment (oral aspirin 300 mg loading and 75 mg daily, with or without clopidogrel 300 mg loading and 75 mg daily, for 1 month) or anticoagulation as appropriate, long-term antiplatelet treatment (75 mg aspirin or clopidogrel daily), blood-pressure lowering (usually starting with perindopril and indapamide), and lipid-lowering (usually with atorvastatin 40–80 mg). Patients who were first seen in the emergency department or elsewhere before referral to the study clinic were initially managed acutely according to the judgment of the treating doctor.

Patients were followed up at face-to-face visits at 1 month, 6 months, and 1, 5, and 10 years, to identify recurrent events. Recurrent events were also identified acutely by ongoing daily ascertainment in the OXVASC population, including a final computerised search of primary care practice diagnostic codes at the end of follow-up (on Oct 1, 2018). Recurrent stroke was defined according to the WHO definition (ie, requiring symptoms lasting at least 24 h). Whenever a recurrent stroke occurred, patients were seen by a study doctor and cases were reviewed by the senior neurologist (PMR). If a patient moved out of the area or to another primary care practice, follow-up was done by phone. All patients were also flagged with the Office for National Statistics such that all death certificates were obtained, and additional information was gathered on all out-of-hospital deaths from the Coroner's Office.

### Statistical analysis

Analyses included all patients with their first (in the study period) classic TIA, non-consensus TIA, or minor ischaemic stroke (NIHSS <5).^37^ Analyses were done with SPSS, version 25. Missing data are reported and no data were imputed.

Age-specific incidence rates (per 1000 population per year) were calculated for classic TIA and non-consensus TIA. Since comparisons were within-population, rates were not standardised. We calculated 95% CIs using Poisson distribution or normal approximation, as appropriate.

We compared baseline characteristics, investigations (including acute lesions on diffusion-weighted imaging and site-specific ≥50% arterial stenosis on magnetic resonance angiography or CT angiography), and outcomes between classic TIA, non-consensus TIA, and minor ischaemic stroke. Continuous variables were expressed as mean (SD) and categorical variables as counts and frequencies. Continuous variables were compared by ANOVA and categorical variables with either the χ^2^ test or Fisher's exact test (when the expected cell frequency was <5). We deemed p values less than 0·05 significant. We also calculated ABCD2 and Essen stroke risk scores and compared these between groups.^25^, ^42^

Cumulative risks of recurrent stroke during follow-up (7-day, 90-day, and 10-year risks) were estimated by Kaplan-Meier analysis, censored at the time of the outcome event, death, or end of follow-up (Oct 1, 2018), and risks compared by the log-rank test. Analysis was based on risk from the time of the first ever TIA or stroke in the study period (index event). The stroke risk in this analysis included some patients who only presented to medical attention after they had the stroke but who reported a recent (<90 days) TIA. However, since an unknown number of patients will have had a TIA but not sought medical attention and not subsequently had a stroke, analyses were repeated including only patients who sought medical attention after the index event (ie, excluding recurrent strokes that occurred before seeking attention).

The analysis of 7-day and 90-day risks of stroke from the time of seeking medical attention was also stratified by two potentially confounding factors, delay to presentation (same day *vs* delay ≥1 day) and early antiplatelet treatment (started on or before first presentation to medical attention *vs* delayed), both of which are very strongly related to early risk of recurrent stroke.^2^, ^34^ Using contemporaneous OXVASC data for age-specific and sex-specific incidence of stroke in the underlying study population, we also calculated the extent to which 7-day and 90-day stroke risks from time of seeking medical attention after a non-consensus TIA were greater than those expected based on the background population stroke incidence rate.

### Role of the funding source

The funder of the study had no role in study design, data collection, data analysis, data interpretation, or writing of the report.

## Results

**Figure 1:**
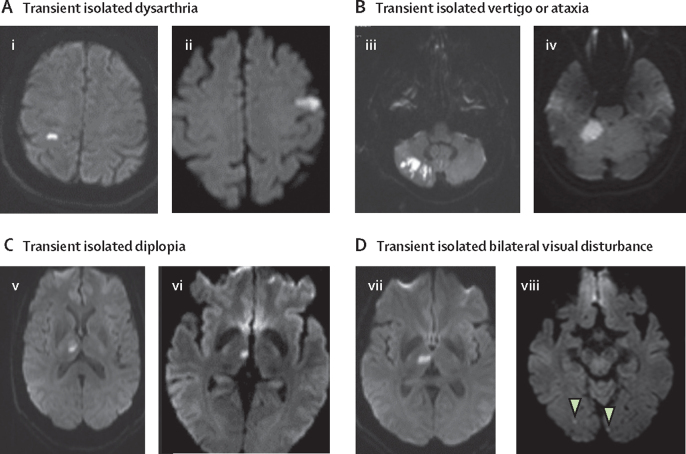
MRIs of sudden onset monosymptomatic non-consensus TIAs in eight different patients with a causative ischaemic lesion (A) Two patients with transient isolated dysarthria. (i) 59-year-old patient who had 30 s episode of slurred speech; examination normal; MRI shows restricted diffusion in right post-central gyrus; MRA normal; grade 2 shunt on bubble TCD. (ii) 64-year-old patient who had sudden onset isolated slurred speech for 12 h, which fully resolved; examination normal next day; MRI shows restricted diffusion in left parietal cortex; MRA normal; grade 2 shunt on bubble TCD. (B) Two patients with transient isolated vertigo or ataxia. (iii) 65-year-old patient with sudden onset isolated vertigo, which lasted 3 min; several recurrences over next few hours; examination showed nystagmus on left lateral gaze; CT brain scan in emergency department was normal; all symptoms resolved after 6·5 h; no hearing loss or tinnitus, but reported episodes of isolated unsteadiness lasting a few seconds, occurring daily for several weeks; MRI shows partial right PICA territory infarct, with tight stenosis of the proximal right vertebral artery on MRA. (iv) 45-year-old patient, heavy smoker, with sudden onset of rotatory vertigo and nausea lasting 12 h; examination normal the following day; MRI shows restricted diffusion in right cerebellum; MRA was normal. (C) Two patients with transient isolated double vision. (v) 64-year-old patient with sudden onset of double vision lasting 30 min; could see normally if covered either eye; saw an optician the following day and no abnormalities were detected; MRI shows restricted diffusion in right thalamus; MRA normal; grade 2 shunt on bubble TCD. (vi) 58-year-old patient woke up with diplopia, with one image diagonally above the other, which resolved on closing either eye; lasted 90 min but had mild headache for 24 h; past history of anxiety and migraine; examination normal; MRI shows restricted diffusion in right thalamus. (D) Two patients with transient isolated bilateral visual disturbance. (vii) 61-year-old patient with sudden onset blurring of the whole visual field; vision was not double; symptoms fully resolved after 30 min; examination normal; MRI shows restricted diffusion in right thalamus; MRA normal; grade 2 shunt on bubble TCD. (viii) 74-year-old with sudden onset blurring of whole visual field lasting 15 min; examination normal; MRI shows bilateral restricted diffusion in occipital lobes (arrows); CT angiography showed bilateral vertebral artery stenosis. MRA=magnetic resonance angiography. TCD=transcranial doppler. PICA=posterior inferior cerebellar artery.

Between April 1, 2002, and March 31, 2018, 2878 patients were identified with minor ischaemic stroke (n=1287), classic TIA (n=1021), or non-consensus TIA (n=570). Of the 570 patients with non-consensus TIAs, 210 reported isolated vertigo or isolated ataxia, 157 isolated sensory loss, 99 bilateral decreased vision without positive symptoms, 57 isolated dysarthria, and 47 isolated diplopia (figure 1).

Follow-up was to Oct 1, 2018 (median 5·2 [IQR 2·6–9·2] years). The annual incidence of non-consensus TIA was 51·0 (95% CI 46·9–55·3) cases per 100 000 population, which was lower than that of classic TIA (91·3 [95% CI 85·8–97·1] cases per 100 000 population), but incidence of both types of TIA increased steeply with age (appendix pp 7–8). Patients with non-consensus TIA were younger than were those with classic TIA (mean age 68·5 [SD 14·1] years *vs* 72·9 [13·6] years; p<0·0001; table 1), had fewer vascular risk factors and a lower frequency of previous stroke (24 [4%] *vs* 69 [7%]; p=0·038), and were less likely to be taking antithrombotic drugs before the index event (177 [31%] *vs* 379 [37%]; p=0·015).

**Table 1:** Baseline characteristics and 7-day and 90-day stroke risk in non-consensus TIA, classic TIA, and minor stroke

		**Non-consensus TIA (n=570)**	**Classic TIA (n=1021)**	**Minor stroke (NIHSS <5; n=1287)**	**p value (non-consensus TIA *vs* classic TIA)**	**Age-adjusted p value**
**Demographics**						
Age, years		68·5 (14·1)	72·9 (13·6)	72·4 (13·9)	<0·0001	..
Sex		..	..	..	0·87	0·57
	Male	281 (49%)	499 (49%)	664 (52%)	..	..
	Female	289 (51%)	522 (51%)	623 (48%)	..	..
**Clinical characteristics**						
Comorbidities						
	Hypertension	287 (50%)	558 (55%)	715 (56%)	0·099	0·99
	Diabetes	54 (9%)	116 (11%)	183 (14%)	0·24	0·40
	Hyperlipidaemia	151 (26%)	300 (29%)	358 (28%)	0·22	0·43
	Atrial fibrillation*	63 (11%)	186 (18%)	219 (17%)	<0·0001	0·010
	Current smoker	74 (13%)	136 (13%)	241 (19%)	0·85	0·095
	Coronary heart disease	66 (11%)	193 (19%)	250 (19%)	<0·0001	0·015
	Peripheral vascular disease	21 (4%)	53 (5%)	83 (6%)	0·17	0·45
	Previous stroke	24 (4%)	69 (7%)	124 (10%)	0·038	0·12
Medication before event						
	Antithrombotic	177 (31%)	379 (37%)	488 (38%)	0·015	0·45
	Antihypertensive	281 (49%)	555 (55%)	732 (57%)	0·053	0·99
	Statin	148 (26%)	315 (31%)	359 (28%)	0·040	0·29
**Stroke risk from time of index event**						
7-day events		38/570	90/1021	42/1287	..	..
	7-day risk	6·7% (5·7–8·7)	8·8% (7·0–10·6)	3·3% (2·3–4·3)	0·12	0·14
90-day events		59/570	118/1021	93/1287	..	..
	90-day risk	10·6% (7·8–12·9)	11·6% (9·6–13·6)	7·2% (5·8–8·6)	0·43	0·55
**Stroke risk from time of seeking medical attention**†						
7-day events		15/521	53/986	38/1346	..	..
	7-day risk	2·9% (1·5–4·3)	5·5% (5·0–6·8)	2·8% (1·8–3·8)	0·025	0·025
90-day events		19/521	70/986	83/1346	..	..
	90-day risk	3·6% (2·1–5·1)	7·1% (5·5–8·7)	6·2% (4·8–7·6)	0·0070	0·019

Data are mean (SD), n (%), n/N, or % (95% CI). NIHSS=National Institutes of Health Stroke Score. TIA=transient ischaemic attack.

* Patients with known atrial fibrillation before diagnosis or new atrial fibrillation at baseline assessment.

† The analysis of stroke risk from time of seeking medical attention excludes 25 patients who presented with a major stroke and is stratified by the nature of the presenting event rather than the index event.

On baseline investigation, patients with classic TIA and non-consensus TIA had similar rates of atrial fibrillation on 5-day ambulatory monitoring (26 of 413 [6%] *vs* 14 of 198 [7%]; p=0·70), patent foramen ovale (84 of 263 [32%] *vs* 46 of 115 [40%]; p=0·16), and any arterial stenosis 50% or greater on cerebrovascular imaging (249 of 896 [28%] *vs* 126 of 467 [27%]; p=0·75). However, more patients with non-consensus TIA had arterial stenosis of 50% or greater in the posterior circulation than did those with classic TIA (84 of 467 [18%] *vs* 80 of 896 [9%]; odds ratio [OR] 2·21, 95% CI 1·59–3·08; p<0·0001), particularly those with syndromes (eg, vertigo or ataxia, diplopia, and bilateral decreased vision) that localise to the posterior circulation (table 2).

**Table 2:** Distribution of ≥50% arterial stenosis according to vascular territory on baseline investigation in patients who underwent vascular imaging

		**Intracranial or extracranial arterial stenosis ≥50%**		**p value**
		Any anterior circulation	Any posterior circulation	
Classic TIA		193/896 (22%)	80/896 (9%)	<0·0001
Non-consensus TIAs		55/467 (12%)	84/467 (18%)	0·0001
	Isolated vertigo or ataxia*	23/169 (14%)	35/169 (21%)	..
	Isolated diplopia*	7/45 (16%)	14/45 (31%)	..
	Bilateral decreased vision*	9/83 (11%)	15/83 (18%)	..
	Isolated dysarthria†	5/45 (11%)	6/45 (13%)	..
	Unilateral sensory disturbance†	9/125 (7%)	13/125 (10%)	..

Data are n/N (%). 22 of 896 patients with classic TIA and 13 of 467 with non-consensus TIA had both anterior and posterior circulation stenosis. TIA=transient ischaemic attack.

* Events likely to be due to posterior circulation ischaemia.

† Events due to either posterior or anterior circulation ischaemia.

From 2009 onwards, MRI was done in 349 patients with classic TIA versus 228 patients with non-consensus TIA. Acute ischaemic lesions on diffusion-weighted imaging were more frequent after classic TIA versus non-consensus TIA (58 of 349 [17%] *vs* 21 of 228 [9%]; p=0·013), although the frequency was similar for classic TIA affecting the posterior circulation (seven of 102 [7%]). In patients with non-consensus TIA, positive lesions on diffusion-weighted imaging occurred in seven of 96 patients with isolated vertigo or ataxia, three of 66 with isolated sensory loss, five of 14 with isolated dysarthria, four of 21 with isolated diplopia, and two of 31 with bilateral decreased vision (examples shown in figure 1).

**Figure 2:**
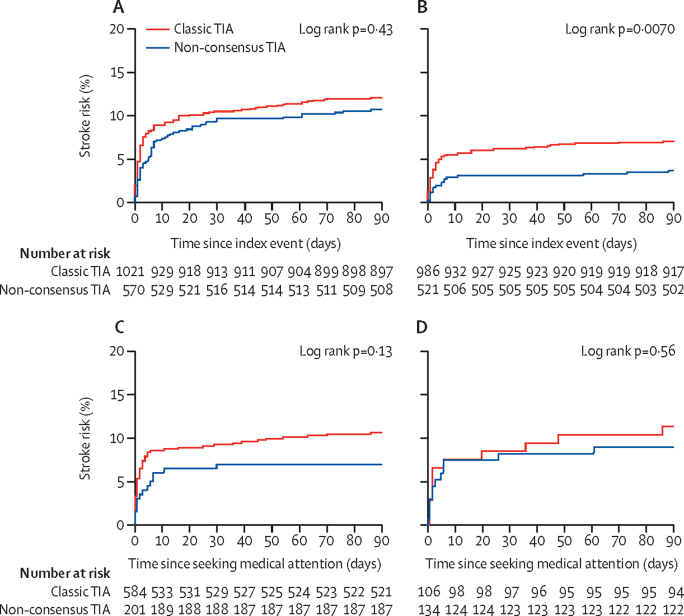
90-day stroke risk in patients with classic TIA and non-consensus TIA Plots show 90-day stroke risk from time of index event (A), from time of seeking medical attention (B), from time of seeking medical attention in patients who sought attention on the day of the index event (C), and from time of seeking medical attention in patients in whom antiplatelet treatment was not started at initial presentation (D). TIA=transient ischaemic attack.

**Figure 3:**
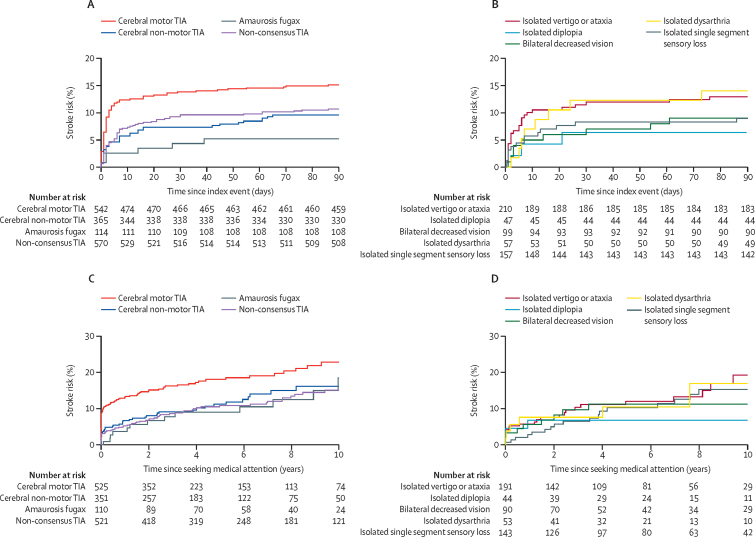
90-day stroke risk from time of index event in patients with classic TIA and non-consensus TIA Plots show 90-day stroke risk in patients with classic TIA and non-consensus TIA (A) and non-consensus TIA stratified by symptoms (B), and 10-year stroke risk from time of seeking medical attention in patients with classic TIA and non-consensus TIA (C) and non-consensus TIA stratified by symptoms (D). TIA=transient ischaemic attack.

During 17 009 person-years of follow-up, 577 patients had their first recurrent stroke after the index event, with 279 happening after an index minor stroke, 199 after an index classic TIA, and 99 after an index non-consensus TIA. From the time of the index event, patients with non-consensus TIA had a similar 90-day stroke risk to patients with classic TIA (10·6% [95% CI 7·8–12·9] *vs* 11·6% [9·6–13·6]; hazard ratio [HR] 0·87, 95% CI 0·64–1·19; p=0·43; table 1; figure 2) and a similar 90-day risk of stroke and acute cardiac events such as myocardial infarction and sudden cardiac death (11·2% [95% CI 8·6–13·7] *vs* 12·9% [10·9–14·8]; p=0·29). The 90-day stroke risk after a non-consensus TIA was lower than after a classic TIA with motor symptoms (10·6% [95% CI 7·8–12·9] *vs* 14·4% [11·5–17·3]; p=0·030), similar to risk after a classic cerebral TIA without motor symptoms (8·9% [6·0–11·8]; p=0·44), and higher than after amaurosis fugax (4·3% [0·6–8·0]; p=0·042; figure 3). The numbers of cases with each individual non-consensus TIA syndrome were too small to estimate risks reliably, but there was no heterogeneity in 90-day stroke risk (p_heterogeneity_=0·36).

98 patients had their first recurrent stroke after the index event before seeking medical attention, of which six were after an index minor stroke, 47 were after an index classic TIA, and 45 were after an index non-consensus TIA, with a higher rate of recurrent stroke after non-consensus TIA than after classic TIA (45 of 570 [8%] *vs* 47 of 1021 [5%]; OR 1·77, 95% CI 1·16–2·71; p=0·007). After excluding such events, 479 first recurrent strokes occurred during the 10 years after seeking medical attention, of which 273 were after a presenting minor stroke, 147 were after a presenting classic TIA, and 59 were after a presenting non-consensus TIA. The 10-year risk of intracranial haemorrhage was 2·4% (95% CI 0·0–4·8 [14 of 1021]) among patients with classic TIA versus 0·8% (95% CI 0·0–1·6 [four of 570]) in patients with non-consensus TIA (p=0·16).

The 90-day stroke risk after seeking medical attention was higher after classic TIA (7·1% [95% CI 5·5–8·7]) than after non-consensus TIA (3·6% [2·1–5·1]; HR 2·0, 95% CI 1·2–3·3; p=0·0070; table 1, figure 2), and was similar to that after minor stroke (6·2% [95% CI 4·8–7·6]; p=0·32; table 1). However, patients with non-consensus TIA were less likely to seek medical attention on the day of the event than were those with classic TIA (336 of 570 [59%] *vs* 768 of 1021 [75%]; OR 0·47, 95% CI 0·38–0·59; p<0·0001; appendix p 8) and were more likely to delay seeking medical attention for at least 3 days (191 of 570 [34%] *vs* 170 of 1021 [17%]; OR 2·30, 95% CI 1·78–3·07; p<0·0001). After adjusting for the greater delay in seeking medical attention after non-consensus TIA versus classic TIA, there was no longer any difference in the early risk of stroke after seeking attention (7-day adjusted HR 0·57, 95% CI 0·27–1·22; p=0·15; 90-day adjusted HR 0·64, 95% CI 0·36–1·12; p=0·12). Findings were similar after further adjustment for age, sex, and baseline vascular risk factors (7-day adjusted HR 0·75, 95% CI 0·42–1·36; p=0·35; 90-day adjusted HR 0·67, 95% CI 0·40–1·13; p=0·14). Moreover, the 7-day risk of stroke after seeking attention for non-consensus TIA (2·9% [95% CI 1·5–4·3]; table 1) was considerably higher than the expected background population stroke risk (age and sex adjusted relative risk [RR] 203, 95% CI 113–334), particularly if patients sought attention on the day of the index event (5·0%, 95% CI 2·1–7·9; RR 300, 95% CI 137–569).

ABCD2 scores were lower in patients with non-consensus TIA (mean 2·8) than in those with classic TIA (4·1; p<0·0001; appendix p 9). The score predicted 90-day stroke risk after seeking medical attention after classic TIA (9·1%, 95% CI 6·9–11·3 [59 of 645] for an ABCD2 score ≥4 *vs* 2·2%, 0·6–3·8 [seven of 320] for an ABCD2 score <4; p<0·0001), but not after non-consensus TIA (2·7%, 95% CI 0·2–5·2 [four of 147] *vs* 3·8%, 1·8–5·8 [14 of 367]; p=0·53).

Patients with non-consensus TIA were less likely than were those with classic TIA to be treated (ie, treatment initiated or continued) with antiplatelets at first presentation to medical attention. 134 (26%) of 521 patients with non-consensus TIA were untreated versus 106 (11%) of 986 with classic TIA (p<0·0001). In untreated patients, the 90-day risk of stroke from time of seeking medical attention was similar after non-consensus TIA versus classic TIA (9·8%, 95% CI 4·7–14·9 [13 of 132] *vs* 11·1%, 5·2–17·0 [12 of 110]; HR 0·89, 95% CI 0·40–1·90; p=0·77; figure 2). Patients with non-consensus TIA who received antiplatelets (ie, treatment was initiated or continued) had a lower 90-day risk of stroke after seeking attention than did those who were not treated (1·3%, 95% CI 0·1–2·5 [five of 388] *vs* 9·8%, 4·7–14·9 [13 of 132]; p<0·0001), and this difference was seen both in patients referred directly to the study TIA or stroke clinic (two of 248 [1%] *vs* seven of 101 [7%]; p=0·0020) and in those seen first by other services (two of 173 [1%] *vs* six of 31 [19%]; p<0·0001).

Prescription of drugs for secondary prevention remained higher at 1-month follow-up for patients with classic TIA than for those with non-consensus TIA. For example, antithrombotics were prescribed for 955 (97%) of 986 patients with classic TIA versus 422 (81%) of 521 with non-consensus TIA (p<0·0001). Antihypertensives were prescribed for 756 (77%) of 986 patients with classic TIA versus 343 (66%) of 521 with non-consensus TIA (p<0·0001). Statins were prescribed for 791 (80%) of 986 patients with classic TIA versus 329 (63%) of 521 with non-consensus TIA (p<0·0001). However, these differences were accounted for mainly by low-risk patients (ie, those with an Essen score ≤2), with less disparity seen in high-risk patients (appendix p 10).

**Figure 4:**
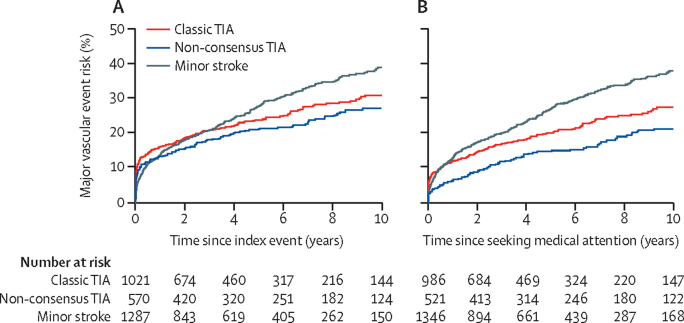
10-year risk of all major vascular events in patients with non-consensus TIA, classic TIA, and minor ischaemic stroke Plots show 10-year risk from time of index event (A) and from time of seeking medical attention (B). TIA=transient ischaemic attack.

The 10-year risk of stroke after seeking medical attention for non-consensus TIA was lower than that after classic TIA (15·0% [95% CI 11·1–18·9] *vs* 20·1% [16·6–23·6]; HR 1·5, 95% CI 1·1–2·0; p=0·010), but the difference between groups diminished after adjusting for time to seek medical attention (adjusted HR 0·78, 95% CI 0·54–1·11; p=0·17) and for age, sex, and vascular risk factors (0·83, 0·58–1·20; p=0·33). The 10-year risk of all major vascular events was similar for patients with non-consensus TIA and for those with classic TIA (27·1% [95% CI 22·8–31·4] *vs* 30·9% [27·2–33·7]; p=0·12; age and sex adjusted HR 1·06, 0·85–1·31; p=0·60; figure 4).

The 10-year stroke risk was similar for patients with non-consensus TIA, classic cerebral TIA without motor symptoms, and patients after amaurosis fugax, and did not differ between the individual non-consensus syndromes (p_heterogeneity_=0·38; figure 3). Patients with non-consensus TIA had lower Essen stroke risk scores than did those with classic TIA. 187 (36%) of 521 patients with non-consensus TIA and 496 (50%) of 986 with classic TIA had an Essen score greater than 2 (p<0·0001), but the Essen score predicted 10-year stroke risk in both groups (appendix p 11).

In patients with negative findings on diffusion-weighted imaging in the MRI cohort (ie, 291 patients with classic TIA and 207 patients with non-consensus TIA), the long-term stroke risk after seeking medical attention (limited to 5 years because of shorter available follow-up) in those with non-consensus TIA was similar to the risk in patients with classic TIA (5·3% [95% CI 1·7–8·8] *vs* 7·6% [3·1–12·0]; p=0·46).

## Discussion

The evidence base for diagnosis of TIAs is limited. Since acute ischaemic lesions on diffusion-weighted imaging are present in relatively few patients with a definite TIA,^19^, ^20^ symptomatic diagnosis remains the mainstay of clinical practice. However, the widely accepted high-level definition of TIA as a sudden onset, focal neurological deficit of presumed vascular origin lasting less than 24 h^8^, ^9^ provides no guidance on which symptoms are likely to be vascular in origin. Interobserver agreement between clinicians for diagnosis of classic TIA is good,^11^, ^14^ but there is only moderate agreement when all TIA referrals are included, even when written scenarios are used.^11^, ^12^, ^14^, ^43^, ^44^ This clinical disagreement, which is driven mainly by non-consensus TIAs,^14^, ^44^, ^45^, ^46^, ^47^ undermines effective management in the absence of a gold standard diagnostic test.

The low prevalence of acute ischaemic lesions on diffusion-weighted imaging, particularly in posterior circulation TIAs, means that there are currently only two indicators of the likely nature of non-consensus TIAs—ie, the risk of subsequent stroke, and the frequency of vascular pathological findings on investigation. In our prospective population-based study of patients seeking medical attention with suspected TIAs or stroke, we noted that the risk of recurrent stroke after non-consensus TIA was much higher than age-matched or sex-matched stroke incidence rates in the underlying population, overlapping with the risk after non-motor classic TIAs, and that frequencies of vascular pathological findings were also similar to those in patients with classic TIA.

Our analysis of the short-term risk of recurrent stroke was stratified by two key confounders, delay to presentation and early antiplatelet treatment, both of which strongly affect the early risk of recurrent stroke.^5^, ^34^ Patients with non-consensus TIAs delayed seeking medical attention compared with those with classic TIAs, but the risks of recurrent stroke were similar for all patients who sought medical attention on the day of the index event (whether classic or non-consensus TIA). Patients with non-consensus TIAs were also less likely to be given immediate antiplatelet treatment when they did seek attention, and the high early risk of stroke in untreated patients was reminiscent of the early risk reported after classic TIAs in the era before urgent treatment was given.^2^ The risk of early recurrent stroke after non-consensus TIA was much lower in patients who were given immediate antiplatelet treatment, consistent with the effects of aspirin on early risk of recurrent stroke in randomised trials^5^ and with other studies of acute treatment.^3^, ^4^

The ABCD2 score was lower in patients with non-consensus TIA than in those with classic TIA, which was as expected based on the younger age of these patients, their absence of motor weakness, and fewer cases with speech disturbance. Use of a high ABCD2 score as an inclusion criterion in randomised trials of acute treatment for TIA indirectly excludes patients with non-consensus TIAs. Essen scores for longer term stroke risk were also lower in patients with non-consensus TIAs, partly reflecting the younger age, but 10-year risk of stroke or acute coronary events was similar to risk after classic TIAs when stratified by the Essen score.

Baseline rates of atrial fibrillation, patent foramen ovale, and stenosis of 50% or greater of the intracranial or extracranial arteries were similar in patients with non-consensus TIA versus those with classic TIA, providing further evidence that non-consensus events do represent TIAs. That those non-consensus TIAs with symptoms usually localised to the posterior circulation had high rates of posterior circulation arterial stenosis is also supportive. Indeed, rates of stenosis in posterior circulation non-consensus TIAs were higher than in posterior circulation classic TIAs.

Our study has several strengths. First, we had near-complete ascertainment of all patients who sought medical attention with possible TIA or stroke in Oxfordshire, UK, which was facilitated by referral from the collaborating emergency department and by primary care doctors, who were regularly reminded to refer all patients with a possible TIA or minor stroke to the daily urgent assessment study clinic, even if symptoms were atypical. Second, we prospectively classified clinical events at first presentation (before recurrent events). Third, patients were assessed acutely by skilled stroke researchers so the most accurate description of events possible could be obtained, before symptoms were forgotten. Fourth, we had high rates of long-term face-to-face follow-up, supplemented by access to medical records and centralised diagnostic codes. Finally, the most important difference of our study from previous studies is the focus on the definable subset of non-consensus transient, focal, negative monosymptomatic events, rather than the catch-all and less clinically generalisable definitions of transient neurological attacks or atypical TIAs (appendix p 2), which include many different syndromes.

Our study has some limitations. First, it was done in Oxfordshire, UK, in a mainly white European population, and so results might not be generalisable to other settings. Second, although diagnostic classification was prospective and, therefore, blind to the occurrence of subsequent recurrent events, and it was based on prespecified syndrome descriptions, some element of subjective clinical judgment is inevitable. Moreover, the designation of an isolated symptom is dependent on the patient's memory and the quality of recording a medical history. However, most patients were seen acutely, and a detailed clinical history was obtained by a dedicated clinical researcher rather than by a busy non-study clinician. Moreover, prespecified syndromic diagnosis is the basis of much medical practice and, thus, the prognosis of non-consensus TIA is likely to be broadly generalisable. Third, routine investigation with brain MRI was restricted to patients enrolled at a later stage in the cohort, but this method was then used consecutively, with no difference in imaging rates between classic TIAs and non-consensus TIAs. Fourth, although 570 patients with non-consensus TIAs with isolated negative focal symptoms is, to our knowledge, the largest cohort assembled and followed up to date, further studies and pooled analyses would allow risk estimates to be calculated more reliably in individual clinical syndromes. Fifth, our analysis of stroke risk from the time of the index TIA included some patients who only presented to medical attention after they had a stroke, but it was unable to include an unknown number of patients who had a TIA but who did not seek medical attention and did not subsequently have a stroke. However, this bias applies to both classic TIAs and non-consensus TIAs, and we also reported prospective risks of stroke after seeking medical attention. Moreover, the 7-day stroke risk after seeking attention for non-consensus TIAs, if the patient sought attention on the day of the event, was high after non-consensus TIA (5%), 300 times higher than the expected background risk, and the risk in all non-consensus TIAs was similar to that after classic TIA without motor symptoms. Sixth, patients were classified based on the first event in the study period, but some patients subsequently had multiple events of different types, sometimes including both non-consensus and classic TIAs. Finally, we did not report data for other atypical TIA-like symptoms (eg, events with progressive, positive, or non-focal symptoms, including amyloid spells) or the number of patients with TIA mimics and non-vascular diagnoses. Patients with these events were identified and will be reported separately.

Our findings have several implications for clinical practice. First, they should help to improve reproducibility of clinical diagnoses of TIA and inform guidelines on diagnostic criteria. Second, patients with non-consensus TIAs should not be falsely reassured about the nature of their symptoms or about a benign prognosis but should be treated similarly to patients with classic TIA. The less steep increase in incidence of non-consensus TIAs versus classic TIAs with age (appendix pp 7–8) suggests that events in older patients were either being misdiagnosed in primary care and not referred or that older patients were not seeking medical attention. Third, patients with non-consensus TIAs should not be routinely excluded from clinical trials and other research studies. Fourth, in view of the substantial number of recurrent strokes that occurred before patients sought medical attention after both non-consensus TIAs and classic TIAs, public education is needed to encourage people to seek medical attention after sudden onset untoward neurological symptoms. Fifth, although designation of non-consensus TIAs as definite cerebrovascular events is justified by our findings, it will increase overall TIA diagnoses by about 50%, with implications for imaging capacity and other aspects of service provision. However, the health economic case for urgent and intensive management of TIAs is strong.^47^ Finally, a broader definition of TIA is likely to lead to more referrals of TIA mimics for assessment. However, some of the non-consensus syndromes (eg, isolated diplopia and isolated dysarthria) have relatively few mimics and, although the differential diagnosis of isolated vertigo includes initial presentations of several common conditions (eg, benign positional vertigo, Ménière's disease, and labyrinthitis), most cases can be reliably differentiated and merit assessment anyway.

In conclusion, our findings show that several common, sudden onset, transient, isolated, negative, focal neurological symptoms (ie, non-consensus TIAs) are associated with high short-term and long-term risks of stroke and other acute vascular events, have similar rates of vascular pathological findings on baseline investigation to classic TIAs, and should be treated in the same way as are classic TIAs. That small ischaemic events might often cause short-lived and isolated neurological symptoms is not unexpected, since brain imaging in asymptomatic older individuals typically shows ischaemic lesions that have been completely silent.

## Data sharing

Requests for access to data reported in this Article will be considered by PMR.
