# Supplementary material for: Diagnosis of non-consensus transient ischaemic attacks with focal, negative, and non-progressive symptoms: population-based validation by investigation and prognosis
Source: Lancet. 2021 Mar 6;397(10277):902–12. doi: 10.1016/S0140-6736(20)31961-9 (PMC7938377; doi:10.1016/S0140-6736(20)31961-9)
Supplement: Supplementary appendix [file mmc1.pdf]

THE LANCET

Supplementary appendix

This appendix formed part of the original submission and has been peer reviewed. We post it as supplied by the authors.

Supplement to: Tuna MA, Rothwell PM, on behalf of the Oxford Vascular Study. Diagnosis of non-consensus transient ischaemic attacks with focal, negative, and non-progressive symptoms: population-based validation by investigation and prognosis. *Lancet* 2021; **397:** 902–12.

Webappendix

Web appendix	Title	Page
1	Type of sudden transient neurological symptoms included in OXVASC and previous studies.	2
2	Inclusion/exclusion of non-consensus transient ischaemic attack (TIA) syndromes in those guidelines and reviews on diagnosis of TIA, those studies of diagnostic accuracy, and in those randomised trials of acute treatment that addressed the issue in the publication or protocol.	3
3	Transient Ischaemic Attack studies where syndromic diagnoses were not given or were reported to be based on the original NINDS definition.	3
4	References to web tables 2 & 3.	4
5	Oxford Vascular Study methodology	5-6
6	Age-specific incidence rates (95% CI) of classical TIA and non-consensus TIA in the Oxford Vascular Study population from 2002-2018.	7
7	Comparisons of classical TIA and non-consensus TIA for: (A) Age-specific incidence (95% CI); (B) Time to call for medical attention after the index event.	8
8	The relative distribution of ABCD2 stroke risk scores[24] in patients with classical TIA versus non-consensus TIAs (A) and the distribution of Essen stroke risk score[42] stratified by type of event (B)	9
9	Rates of use of secondary preventive medication at the one-month follow-up in patients with non-consensus TIA versus classical TIA stratified according to the Essen stroke risk score.	10
10	Cumulative stroke risk at 90-days and 10-years after seeking medical attention for non-consensus TIA, classical TIA and minor stroke stratified according to the Essen stroke risk score.	11

**Web table 1. Type of sudden transient neurological symptoms included in OXVASC and previous studies.**			
	TIA	Other Transient Neurological Symptoms	Comments
OXVASC	NINDS-Definition<br>One or more of the following for anterior circulation events: (unilateral motor or sensory symptoms, dysphasia, transient monocular blindness).<br>For posterior circulation events: bilateral (during single attack) motor or sensory symptoms or hemianopia/quadrantanopia; diplopia, vertigo, ataxia, dysphagia or dysarthria if associated with one or more of the above symptoms.<br><br>According to NINDS-definition: vertigo, diplopia, dysarthria in isolation are not consider a TIA	Non-consensus TIA: isolated non-progressive (all symptoms maximal in onset), negative focal symptoms (deficits) lasting < 24h:<br>Isolated vertigo<br>Isolated ataxia (usually unsteadiness)<br>Isolated diplopia<br>Isolated non-dysphasic speech disturbance (dysarthria)<br>Isolated unilateral sensory loss in one body segment only<br>Bilateral reduced/loss vision	Events excluded from current analyses: non-vascular events (TIA/stroke mimics); and Transient Neurological Attacks presenting as positive symptoms (visual, paresthaesia), or progressive symptoms (march), or confusion, unresponsiveness, limb jerks.
Rotterdam [29]	Focal brain symptoms = TIA<br>(one or more of the following: hemiparesis, hemihypesthesia, dysphasia, dysarthria, amaurosis fugax, hemianopia, hemiataxia, diplopia, or vertigo)	Non-focal Transient Neurological Attacks (syncope, confusion, transient global amnesia)<br>Mixed (focal and non-focal) Transient Neurological Attacks.	Events excluded: clear evidence of migraine, epilepsy, Ménière disease, hyperventilation, cardiac syncope, hypoglycaemia, orthostatic hypotension.<br>Single isolated vertebrobasilar symptoms (isolated diplopia, vertigo, or dysphagia) were also excluded (n=12).
Dutch TIA trial [28]	NINDS-definition (as above)	Atypical TIA: disturbance of vision in one or both eyes consisting of flashes, lines, objects, distorted view, tunnel vision image moving on change of posture; alteration of muscle strength consisting of tired or heavy sensation in one or more limbs, either unilateral or bilateral; sensory disturbance alone (unilateral or bilateral) or a gradual spread of sensory symptoms; brainstem symptoms and coordination difficulties consisting of isolated disorders of swallowing or articulation, double vision, dizziness or uncoordinated movements; accompanied symptoms including unconsciousness, limb jerking, tingling of limbs or lips, disorientation, amnesia.	
SOS TIA[30]	NINDS-definition (as above)	Atypical transient symptoms (possible TIA): isolated partial sensory deficit, dysarthria, vertigo/unsteadiness, unusual cortical visual deficit (lone bilateral blindness and bilateral positive visual phenomena) and diplopia.	Cohort of patients with a suspected TIA referred to TIA clinic. A telephone interview prior to evaluation to limit assessment of patients with diagnosis other than TIA.

**Web table 2. Inclusion/exclusion of non-consensus transient ischaemic attack (TIA) syndromes in those guidelines and reviews on diagnosis of TIA, studies of diagnostic accuracy, and in those randomised trials of acute treatment that addressed the issue in the publication or protocol.**							
Study type	Author	Isolated vertigo	Isolated unsteadiness	Isolated diplopia	Isolated dysarthria	Isolated bilateral decreased vision	Isolated unilateral sensory loss
Guidelines	NINDS.,[9] 1975	x	√	x	x	√	√
	Kernan WN et al.,[18] 2014	x	√	x	x	√	√
Reviews	Landi G.,[8] 1992	x	x	x	x	-	x[a]
	GJ Hankey, CP Warlow.,[21] 1994	x	x	x	x	√	√
	Warlow CP, et al., [22] 2008	x	x	x	x	√	√
Diagnostic studies	Kraaijeveld CL, et al.,[10] 1984	x	x	x	x	√	x
	Koudstaal PJ, et al.,[15] 1986	x	-	x	x	-	√
	Ferro JM, et al.,[11] 1996	x	x	x	x	-	√
	Bos MJ, et al.,[29] 2007	x	-	x	x	-	√
Trials	FASTER.,[w1] 2007	x	x	x	√	x	x
	CHANCE.,[w2] 2013	x	-	x	-	x	x
	SOCRATES.,[w3] 2016	x	-	x	-	x	-
	POINT.,[w4] 2018	x	-	x	-	x	x
	TARDIS.,[w5] 2018	x	-	x	-	x	x
	THALES.,[w6] 2019	x	x	x	x	x	x

Symptom not included (x), included (√), not-specified (-), not included if sensory symptoms confined to one limb, or face only (a); w1-w6: web appendix references p7.

**Web table 3. Transient Ischaemic Attack studies where syndromic diagnoses were not given or were said to be based on the NINDS definition.**	
Study type	Author
Guidelines	Feinberg WM et al.[16]
	Easton JD et al.[17]
	American Stroke Association[18], 2009
	European Stroke Organisation[w7], 2008
	National Institute for Care and Health Excellence[w8], 2019
Diagnosis	Castle J et al.[12]
	Caplan LR.[w9]
	Searls DE et al.[w10]
Trials	ESPRIT[w11]., 2006
	INSPiRE-TMS[w12].,2020

References in web appendix p7 (w7-w12)

4. Additional references for web tables 2 & 3

W1. Kennedy J, Hill MD, Ryckborst KJ, et al. Fast assessment of stroke and transient ischaemic attack to prevent early recurrence (FASTER): a randomised controlled pilot trial. *Lancet Neurol*. 2007;**6**:961-969

W2. Wang Y, Pan Y, Zhao X, et al. Clopidogrel with Aspirin in acute minor stroke or transient ischemic attack. *N Engl J Med* 2013; **369**-11-19.

W3. Johnston SC, Amarenco P, Albers GW, et al. Ticagrelol versus aspirin in acute stroke or Transient Ischemic Attack. *N Engl J Med* 2016; **375**:35-43

W4. Johnston SC, Easton JD, Farrant M, et al. Clopidogrel and aspirin in acute ischemic stroke and high-risk TIA. *N Engl J Med* 2018; 379:**215**-225.

W5. Bath PM, Woodhouse LJ, Appleton JP, et al. Antiplatelet therapy with aspirin, clopidogrel, and dipyridamole versus clopidogrel alone or aspirin and dipyridamole in patients with acute cerebral ischaemia (TARDIS): a randomised, open label, phase 3 superiority trial. *Lancet* 2018; **391**: 850–59.

W6. Johnston SC, Amarenco P, Denison H, et al. The acute stroke or transient Ischemic attack treated with ticagrelor and aspirin for prevention of stroke and death (THALES) trial: Rationale and design. *Int J Stroke* 2019; **14**:745-751.

W7. The European Stroke Organisation (ESO) Executive Committee and the ESO Writing Committee. Guidelines for Management of Ischaemic Stroke and Transient Ischaemic Attack 2008. Cerebrovasc Dis 2008;25:457–507.

W8. National Institute for Haealth an Care Excellence (NICE). Stroke and Transient Ischaemic Attack in over 16s: diagnosis and clinical management. Clinical Guideline [NG128]. May 2019 (https:www.nice.org.uk/guidance/ng128)

W9. Caplan LR. Vertebrobasilar territory ischemia: an overview. In: Caplan LR, ed. Posterior Circulation Disease: Clinical Findings, Diagnosis, and Management. Boston, MA: Blackwell Science; 1996:179-197.

W10. Searls DE, Pazdera L, Korbel E, Vysata O, Caplan LR. Symptoms and Signs of Posterior Circulation Ischemia in the New England Medical Center Posterior Circulation Registry. *Arch Neurol.* 2012;69:346–351.

W11. ESPRIT Study Group; Halkes PH, van Gijn J, Kappelle LJ, Koudstaal PJ, Algra A. Aspirin plus dipyridamole versus aspirin alone after cerebral ischaemia of arterial origin (ESPRIT): randomised controlled trial. Lancet 2006; **367**: 1665–73.

W12. Ahmadi M, Laumeier I, Ihl T et al. A support programme for secondary prevention in patients with transient ischaemic attack and minor stroke (INSPiRE-TMS): an open-label, randomised controlled trial. *Lancet Neurol* 2020; **19**: 49–60.

5. Oxford Vascular Study methodology

Study population: The Oxford Vascular Study (OXVASC) is a prospective, population-based cohort study of all incident acute vascular events in all territories (transient ischaemic attack, stroke, acute coronary and peripheral vascular events).[1,2]

During the period of the current substudy, the OXVASC study population consisted of all 92,728 individuals, irrespective of age, registered with 100 general practitioners (GPs) in nine general practices in Oxfordshire, UK. In the UK, general practices provide primary health care for registered individuals and hold a lifelong record of all medical consultations (from the National Health Service [NHS] and private health care), and details of treatments, blood pressure, and investigations. In Oxfordshire, an estimated 97% of the true residential population is registered with a general practice, with most non-registered individuals being young students. All participating practices held accurate age-sex patient registers, and allowed regular searches of their computerised diagnostic coding systems. The practices had all collaborated on a previous population-based study, for which they were originally selected to be representative of the urban and rural mix and the deprivation range of Oxfordshire as a whole.[3] Based on the index of multiple deprivation (IMD), the population was less deprived than the rest of England, but had a broad range of deprivation.

The OXVASC population is 94% white people, 3% Asian, 2% Chinese, and 1% Afro-Caribbean. The proportion of whites is similar to that of the UK as a whole (88% white) and to many other western countries (Australia - 90%; France - 91%; Germany - 93.9%).

Case ascertainment: After a 3-month pilot study, the study started on April 1, 2002, and is ongoing. Ascertainment combined prospective daily searches for acute events (hot pursuit) and retrospective searches of hospital-care and primary-care administrative and diagnostic coding data (cold pursuit).

Hot pursuit was based on:

1. A daily (weekdays only), urgent open-access “TIA clinic” to which participating general practitioners (GPs) and the local accident and emergency department (A&E) send all individuals with suspected TIA or stroke whom they would not normally admit to hospital, with alternative on-call review provision at weekends. Patients too frail to attend are assessed at their residence by a study nurse or doctor.
2. Daily searches and case note review of admissions to the Emergency Assessment Unit, Medical Short Stay Unit, Coronary Care Unit and Cardiothoracic Critical Care Unit, Cardiology, Cardiothoracic, and Vascular Surgery wards, Acute Stroke Unit, Neurology ward and all other general wards when indicated.
3. Daily searches of the local A&E and eye hospital attendance registers.
4. Daily identification via the Bereavement Office of patients dead on arrival at hospital or who died soon after.
5. Daily searches of lists of all patients from the study population in whom a troponin-I level had been requested.
6. Daily assessment of all patients undergoing diagnostic coronary, carotid and peripheral angiography, angioplasty, stenting or vascular surgical procedures in any territory to identify both total burden of vascular invention and any potential missed prior acute events.

Cold pursuit procedures were:

1. Frequent visits to the study practices and monthly searches of practice diagnostic codes.
2. Monthly practice-specific list of all patients admitted to all acute and community NHS hospitals.
3. Monthly listings of all referrals for brain or carotid imaging studies performed in local hospitals.
4. Monthly reviews of all death certificates and coroners reports to review out-of-hospital deaths.
5. Practice-specific listings of all ICD-10 death codes from the local Department of Public Health.

Patients found on GP practice searches who have an event whilst temporarily out of Oxfordshire are included, but visitors who were not registered with one of the study practices are excluded. A study clinician assessed patients as soon as possible after the event in the hospital or at home. Informed consent was sought, if possible, or assent was obtained from a relative.

Data are collected using event-specific forms, for TIA and stroke, acute coronary syndrome or acute peripheral vascular events. Standardised clinical history and cardiovascular examination are recorded. Information recorded from the patient, their hospital records and their general practice records includes

details of the clinical event, medication, past medical history, all investigations relevant to their admission (including blood results, electrocardiography, brain imaging and vascular imaging-duplex ultrasonography, CT-angiography, MR-angiography or DSA) and all interventions occurring subsequent to the event.

If a patient died before assessment, we obtained an eyewitness account of the clinical event and reviewed any relevant records. If death occurred outside the hospital or before investigation, the autopsy result was reviewed. Clinical details are sought from primary care physicians or other clinicians on all deaths of possible vascular aetiology.

All surviving TIA and stroke patients are followed-up face-to-face at 1, 6, 12, 60 and 120 months after the initial event by a research nurse or physician and all recurrent vascular events were recorded together with the relevant clinical details and investigations. If face-to-face follow up is not possible, telephone follow-up is performed or enabled via the general practitioner. All recurrent vascular events that presented to medical attention would also be identified acutely by ongoing daily case ascertainment within OXVASC. If a recurrent vascular event was suspected at a follow-up visit or referred by the GPs to clinic or admitted, the patient was re-assessed and investigated by a study physician.

Definitions of events

Although new definitions for stroke and TIA have been suggested recently,[4,5] in order to enable comparison with previous studies, the classic definitions of TIA and stroke are used throughout.[6] A stroke is defined as rapidly developing clinical symptoms and/or signs of focal, and at time global (applied to patients in deep coma and to those with subarachnoid haemorrhage), loss of brain function, with symptoms lasting more than 24 hours or leading to death, with no apparent cause other than that of vascular origin.[6] A TIA is an acute loss of focal brain or monocular function with symptoms lasting less than 24 hours and which is thought to be caused by inadequate cerebral or ocular blood supply as a result of arterial thrombosis, low flow or embolism associated with arterial, cardiac or haematological disease.[4] All diagnoses were reviewed by a senior neurologist (PMR). With the high rate (97%) of imaging or autopsy in OXVASC, strokes of unknown type were coded as ischaemic.

References

1. Rothwell PM, Coull AJ, Giles MF, et al. Change in stroke incidence, mortality, case-fatality, severity, and risk factors in Oxfordshire, UK from 1981 to 2004 (Oxford Vascular Study). Lancet 2004; 363: 1925–33.
2. Rothwell PM, Coull AJ, Silver LE, Fairhead JF, Giles MF, Lovelock CE, Redgrave JNE, Bull LM, Welch SJV, Cuthbertson FC, Binney LE, Gutnikov SA, Anslow P, Banning AP, Mant D, Mehta Z for the Oxford Vascular Study. Population-based study of event-rate, incidence, case fatality and mortality for all acute vascular events in all arterial territories (Oxford Vascular Study). Lancet 2005; 366: 1773-83.
3. Bamford J, Sandercock P, Dennis M, Burn J, Warlow C. A prospective study of acute cerebrovascular disease in the community: the Oxfordshire Community Stroke Project--1981-86. 2. Incidence, case fatality rates and overall outcome at one year of cerebral infarction, primary intracerebral and subarachnoid haemorrhage. *J Neurol Neurosurg Psychiatry* 1990;53:16-22.
4. Easton JD, Saver JL, Albers GW, et al. Definition and evaluation of transient ischemic attack: a scientific statement for healthcare professionals from the American Heart Association/American Stroke Association Stroke Council; Council on Cardiovascular Surgery and Anesthesia; Council on Cardiovascular Radiology and Intervention; Council on Cardiovascular Nursing; and the Interdisciplinary Council on Peripheral Vascular Disease. The American Academy of Neurology affirms the value of this statement as an educational tool for neurologists. *Stroke* 2009;40:2276-2293.
5. Sacco RL, Kasner SE, Broderick JP, et al. An updated definition of stroke for the 21st century: a statement for healthcare professionals from the American Heart Association/American Stroke Association. *Stroke* 2013;44:2064-2089.
6. Hatano S. Experience from a multicentre stroke register: a preliminary report. *Bulletin of the World Health Organization* 1976;54:541-553.

6. Age-specific incidence rates (95% CI) of classic TIA and non-consensus TIA in the Oxford Vascular Study population from 2002-2018.

Non-consensus TIA

Age	Number in 16 years	Average number at risk per year	Rate (per 100,000)
0-34	10	43396	1.92 (0.92,3.53)
35-44	25	13421	15.52 (10.05,22.91)
45-54	68	12192	46.48 (36.09,58.92)
55-64	108	10290	87.46 (71.75,105.6)
65-74	145	7343	164.56 (138.86,193.63)
75-84	152	4823	262.63 (222.54,307.86)
85+	62	1735	297.79 (228.31,381.75)
**Total**	**570**	**93200**	**50.97 (46.87,55.33)**

Classic TIA

Age	Number In 16 years	Average number at risk per year	Rate (per 100,000)
0-34	9	43396	1.73 (0.79,3.28)
35-44	30	13421	18.63 (12.57,26.59)
45-54	82	12192	56.05 (44.58,69.57)
55-64	123	10290	99.61 (82.79,118.85)
65-74	266	7343	301.87 (266.68,340.42)
75-84	327	4823	565 (505.42,629.68)
85+	184	1735	883.77 (760.68,1021.1)
**Total**	**1021**	**93200**	**91.29 (85.78,97.07)**

7. Comparisons of classic TIA and non-consensus TIA for: (A) Age-specific incidence (95% CI); (B) Time to call for medical attention after the index event.

A)

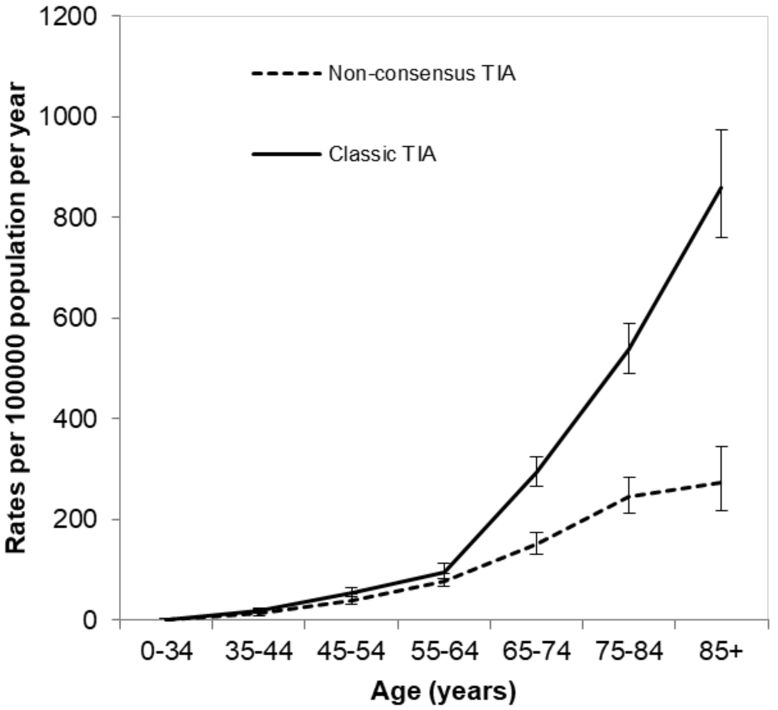

B)

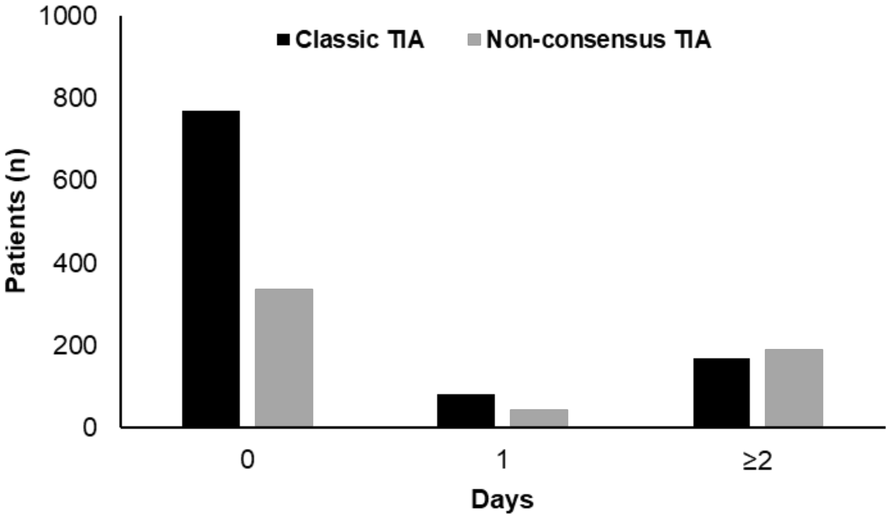

8. The relative distribution of ABCD2 stroke risk scores in patients with classic TIA versus non-consensus TIAs (A) and the distribution of Essen stroke risk score stratified by type of event (B)

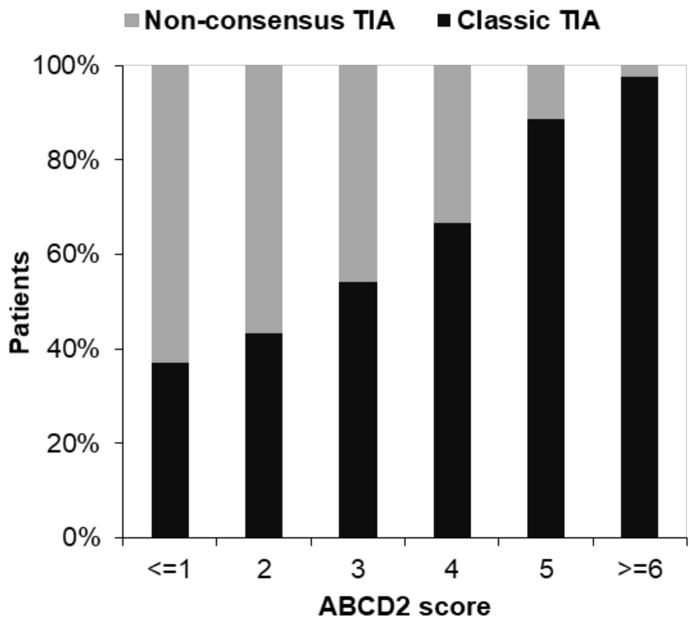

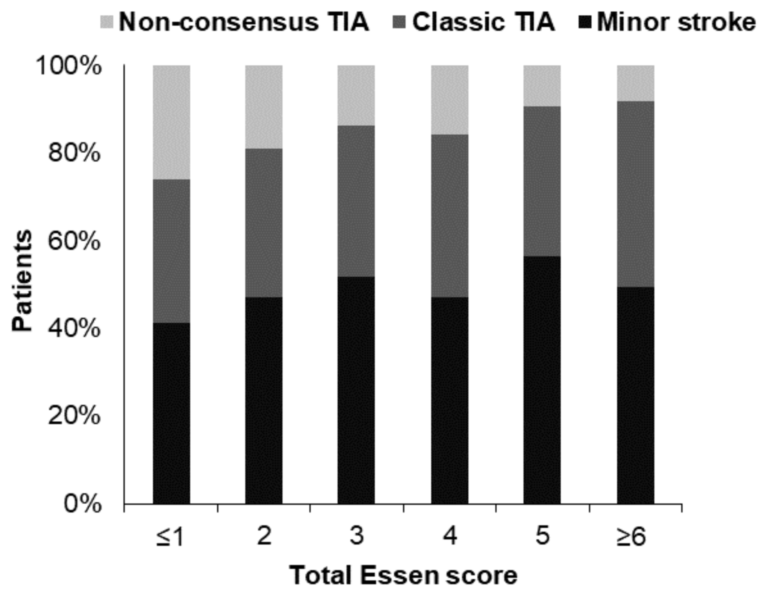

9. Rates of use of secondary preventive medication at one-month follow-up in patients with non-consensus TIA versus classic TIA stratified according to the Essen stroke risk score.

	Non-consensus TIA Essen ≤ 2 (n=334)	Classic TIA Essen score ≤ 2 (n=490)	Non-consensus TIA Essen score >2 (n=187)	Classic TIA Essen score >2 (n=496)
Anti-thrombotic (%)	249/334 (74.6)	478/490 (97.6)	173/187 (92.5)	477/496 (96.2)
Statin (%)	191/334 (57.2)	402/490 (82.0)	138/187 (73.8)	389/496 (78.4)
Anti-hypertensive (%)	171/334 (51.2)	307/490 (62.7)	172/187 (92.0)	449/496 (90.5)

10. Cumulative stroke risk at 90-days and 10-years after seeking medical attention for non-consensus TIA, classic TIA and minor stroke stratified according to the Essen risk score. Patients with recurrent stroke prior to seeking medical attention are excluded.

	90-days		10-years	
Diagnosis	n	Events (% risk)	n	Events (% risk)
Non-consensus TIA	521	19 (3.6)	521	59 (15.0)
Essen score ≤ 2	334	11 (3.3)	334	29 (10.7)
Essen score > 2	187	8 (4.3)	187	30 (25.4)
Classic TIA	986	70 (7.1)	986	146 (20.2)
Essen score ≤ 2	490	25 (5.1)	490	47 (12.2)
Essen score > 2	496	45 (9.1)	496	99 (30.2)
Minor stroke	1346	83 (6.2)	1346	274 (29.5)
Essen score ≤ 2	643	37 (5.8)	643	112 (23.3)
Essen score > 2	703	46 (6.5)	703	162 (37.2)
